# Dihydromyricetin Modulates Nrf2 and NF-κB Crosstalk to Alleviate Methotrexate-Induced Lung Toxicity

**DOI:** 10.3390/ph16040481

**Published:** 2023-03-23

**Authors:** Asmaa I. Matouk, Eman M. Awad, Nashwa F. G. El-Tahawy, Azza A. K. El-Sheikh, Aliaa Anter

**Affiliations:** 1 Department of Pharmacology and Toxicology, Faculty of Pharmacy, Minia University, El-Minia 61511, Egypt; 2 Department of Histology and Cell Biology, Faculty of Medicine, Minia University, El-Minia 61511, Egypt; 3 Basic Health Sciences Department, College of Medicine, Princess Nourah bint Abdulrahman University, P.O. Box 84428, Riyadh 11671, Saudi Arabia

**Keywords:** methotrexate, dihydromyricetin, NF-κB, Nrf2, HO-1, IL-1β, TGF-β1

## Abstract

Background: Methotrexate (MTX) is an effective anticancer, anti-inflammatory, and immunomodulatory agent. However, it induces a serious pneumonitis that leads to irreversible fibrotic lung damage. This study addresses the protective role of the natural flavonoid dihydromyricetin (DHM) against MTX-induced pneumonitis via modulation of Nrf2/NF-κB signaling crosstalk. Methods: Male Wistar rats were divided into 4 groups: control, which received the vehicle; MTX, which received a single MTX (40 mg/kg, i.p) at day 9 of the experiment; (MTX + DHM), which received oral DHM (300 mg/kg) for 14 days and methotrexate (40 mg/kg, i.p) on the 9th day; and DHM, which received DHM (300 mg/kg, p.o) for 14 days. Results: Lung histopathological examination and scoring showed a decline in MTX-induced alveolar epithelial damage and decreased inflammatory cell infiltration by DHM treatment. Further, DHM significantly alleviated the oxidative stress by decreasing MDA while increasing GSH and SOD antioxidant levels. Additionally, DHM suppressed the pulmonary inflammation and fibrosis through decreasing levels of NF-κB, IL-1β, and TGF-β1 while promoting the expression of Nrf2, a positive regulator of antioxidant genes, and its downstream modulator, HO-1. Conclusion: This study identified DHM as a promising therapeutic target against MTX-induced pneumonitis via activation of Nrf2 antioxidant signaling while suppressing the NF-κB mediated inflammatory pathways.

## 1. Introduction

Methotrexate, one of the folate antagonists, has potent anti-inflammatory and anticancer effects [1]. MTX blocks the formation of tetrahydrofolate, an essential substrate in DNA synthesis and repair, leading to inhibition of cellular replication and proliferation. MTX has been effectively used against variety of malignancies, ectopic pregnancies, and autoimmune diseases [2,3]. In addition, it is considered a first-line and gold standard treatment for rheumatoid arthritis (RA) [4]. Other inflammatory diseases, e.g., psoriasis, systemic lupus, multiple sclerosis, and intestinal inflammatory diseases such as Crohn’s disease and ulcerative colitis, effectively respond to MTX treatment [2,5,6]. The anti-inflammatory effects of MTX develop when used in lower doses (7.5–25 mg/week) versus the anticancer doses (1–5 g/week), and are related to the increased intracellular adenosine levels rather than folate antagonism [3,4,7]. Unfortunately, MTX causes multiple organ toxicity, particularly in those with high replicating activity. Patients taking MTX can develop gastrointestinal toxicities, hepatitis, bone marrow suppression, nephrotoxicity, neurotoxicity, and pulmonary toxicity [8,9]. MTX-induced pneumonitis is an unpredictable and serious complication that may occur even with the low MTX doses that are used for management of inflammatory diseases. This may interfere with its clinical use, particularly in RA patients, because they can develop interstitial pneumonitis as a part of their RA disease [10,11]. Despite the fact that it this an infrequent side effect, elderly patients and those with preexisting lung disorders are susceptible to lung damage exacerbated by MTX administration. Clinical symptoms may appear rapidly, after a few days, or may be delayed by more than a year after MTX administration. They include non-productive coughing, fever, and wheezing. However, irreversible pneumonia, breathlessness, and lung fibrosis may develop when taking MTX long-term [12,13,14].

Various theories have explained the pathogenesis of MTX-induced lung injury, including: hypersensitivity reaction with excessive recruitment of eosinophils and neutrophils that damage lung tissues; increased oxidative stress; provoked release of inflammatory cytokines, including IL-1, IL-8, and TNF-α; direct cytotoxic effects on alveolar epithelial cells; and development of pulmonary fibrosis [15,16]. Some antioxidant and anti-inflammatory drugs have been used to counteract lung damage caused by MTX [17,18,19]. 

There is a growing interest in the beneficial effects of dihydromyricetin (DHM), a major flavonoid in Chinese rattan tea. DHM is a polyphenolic compound that was used in traditional Chinese medicine for management of cough, fever, pain, and jaundice, but it is currently used as an antagonist for alcohol intoxication [20]. It has versatile pharmacological actions and high biological safety [21]. DHM has attracted attention for its anticancer activities against liver, lung, and ovarian cancers [22,23,24,25]. It can also be combined with anticancer drugs to decrease the resistance and enhance the curative effects of chemotherapeutic agents [26]. In addition, DHM exhibits cardioprotection, hepatoprotection, nephroprotection, and neuroprotection in multiple pathological models [27,28,29,30]. It also possesses antihyperglycemic and antihyperlipidemic characteristics [31,32]. Most of the beneficial pharmacological effects of DHM are mediated via its ability to inhibit ROS production and to enhance the antioxidant defense system. Further, DHM exerts powerful anti-inflammatory effects by negatively regulating NF-κB, which stimulates the expression of genes involved in inflammation [33]. In a recent study, DHM alleviated the hepatotoxicity induced by MTX via inactivation of TLR4/NF-κB inflammatory signaling pathway [9]. Moreover, several studies have confirmed the role of activation of the nuclear factor erythroid 2–related factor 2 (Nrf2), a regulator of cellular resistance against oxidative stress, in mediating the antioxidant, anti-inflammatory, and antifibrotic effects of DHM [34,35,36]. These beneficial effects rendered DHM a therapeutic target for ameliorating the inflammatory lung damage induced by lipopolysaccharide and sepsis [37,38]. On the contrary, no studies have shown whether DHM could alleviate MTX-induced lung toxicity. The present study investigated the role of DHM in protection of pulmonary damage caused by MTX, and elucidated the role of modulating Nrf2/NF-κB pathways in mediating this effect.

## 2. Results

### 2.1. Dihydromyricetin Decreased MTX-Induced Weight Loss

The effects of single-dose MTX (40 mg/kg) and two weeks of treatment with DHM (300 mg/kg) on the mean body weight of rats are shown in Table 1. At the beginning of the experiment, the mean body weight was comparable in all groups. However, at the end of the experiment, at day 14, the mean body weight of the MTX-treated group had significantly (*p* < 0.05) declined when compared to the control. However, the concurrent treatment with DHM significantly (*p* < 0.05) inhibited the body weight loss induced by MTX. On the other side, administration of DHM alone did not cause significant changes in the body weight when compared to the control group (Table 1).

### 2.2. Dihydromyricetin Attenuated MTX-Induced Histopathological Changes in Lung Tissues

**Figure 1:**
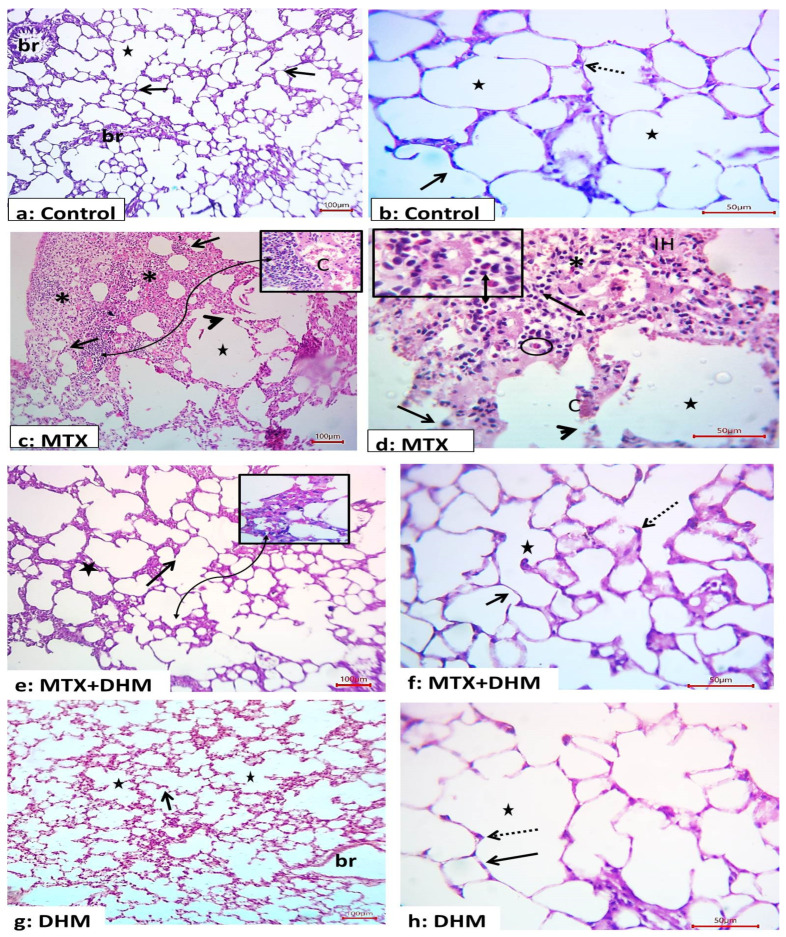
Effect of treatment with dihydromyricetin (DHM, 300 mg/kg, p.o) on methotrexate (MTX, 40 mg/kg, i.p)-induced histopathological changes in lung tissues. Photomicrographs showing lung sections of all groups (n = 6, each): control group (**a**,**b**) showed normal structure of the lung tissues; numerous alveoli (stars) are separated by delicate septa of connective tissue (arrows). Higher magnifications show several connected clear alveoli (stars) lined by pneumocyte type I (arrows) and type II (dashed arrows). MTX group (**c**,**d**) showed disturbed structures; decreased alveoli (stars) with areas of consolidation (*), coalescence of numerous alveoli (stars) with degeneration of their alveolar septa (arrowhead), marked increase in the thickness of inter-alveolar septa (arrows) with inflammatory cell infiltration (curved arrow and insets), and markedly congested blood vessels. Notice interstitial hemorrhage (IH) and inflammatory cells mainly lymphocytes (double head arrows) and eosinophils (circles). The DHM + MTX group (**e**,**f**) showed marked morphological improvement, with few areas showing thickening of the alveolar septa (curved arrow and inset). The DHM group (**g**,**h**) showed normal morphological changes, with numerous alveoli (stars) separated by delicate septa of connective tissue (arrows); H&E (**a**,**c**,**e**,**g**) ×10; scale bar: 100 µm, (**b**,**d**,**f**,**h**) ×40; scale bar: 50 µm; and insets ×100 scale bar: 20 µm.

Administration of MTX (40 mg/kg, i.p) resulted in marked histopathological changes in lung tissues. Lung sections of the MTX-treated group displayed disturbed structures; we observed decreased alveoli with areas of consolidation. Further, the coalescence of numerous alveoli with degeneration of their inter-alveolar septa was observed. Other areas had marked increases in the thickness of the inter-alveolar septa, with interstitial hemorrhage, inflammatory cell infiltration (mainly eosinophils and lymphocytes), and markedly congested blood vessels (Figure 1). Alternatively, treatment with DHM (300 mg/kg, p.o) for 2 weeks showed marked morphological improvement of the previous changes, with few areas that still showing thickening of the alveolar septa (Figure 1). The control and DHM groups showed normal structure of the lung tissues; numerous alveoli (stars) were separated by delicate septa of connective tissue (arrows). The alveoli appear as numerous connected air sacs lined by single layer of pneumocyte type I (numerous and flat) and type II (few and rounded cells) (Figure 1). Scoring of pulmonary histological features, which included alveolar consolidation, thickness of alveolar wall, vascular congestion, interstitial hemorrhage, and inflammatory cell infiltration, confirmed that rats administered MTX developed significant (*p* < 0.05) degenerative changes in their lung tissues when compared to control rats. In contrast, DHM treatment significantly (*p* < 0.05) attenuated these changes and apparently preserved the lung tissues against MTX-induced injury (Table 2). 

### 2.3. Dihydromyricetin Enhanced the Antioxidant Enzymes while Attenuating MDA Levels in Lung Tissues of MTX-Treated Rats

**Figure 2:**
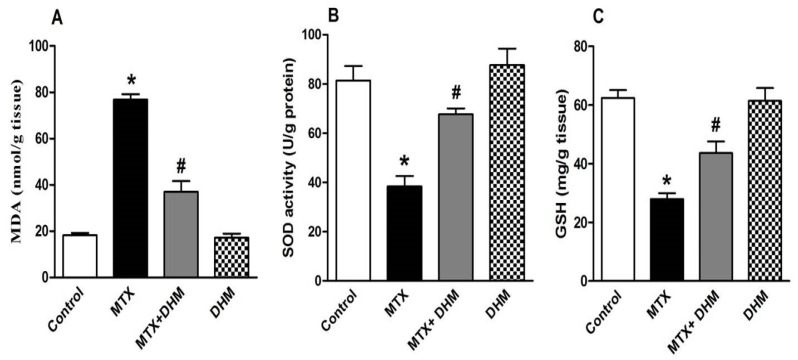
Effects of 2 weeks of treatment with dihydromyricetin (DHM, 300 mg/kg, p.o) on oxidative stress parameters in methotrexate (MTX, 40 mg/kg, i.p)-induced lung toxicity. (**A**) MDA, (**B**) SOD, and (**C**) GSH. Data are presented as means ± SEM (n = 6). * and # indicate significant differences from the control and MTX groups, respectively, at (*p* < 0.05).

An assessment of oxidative stress revealed that the administration of MTX increased ROS production in lung tissues. This effect was confirmed by the significant (*p* < 0.05) elevation in pulmonary levels of MDA, which is usually used as a marker of ROS-dependent lipid peroxidation (Figure 2A). As a consequence, pulmonary levels of the antioxidant enzymes SOD and GSH (Figure 2B,C), respectively, had significantly (*p* < 0.05) declined in the MTX-treated group. Conversely, 2 weeks of treatment with DHM (300 mg/kg, p.o) concurrently with MTX resulted in a significant (*p* < 0.05) alleviation of pulmonary MDA levels (Figure 2A), together with a significant (*p* < 0.05) elevation of SOD and GSH levels (Figure 2B,C), thus indicating decreased oxidative stress in lung tissues that were subject to DHM treatment. On the other hand, the group given DHM (300 mg/kg, p.o) alone for 2 weeks showed non-significant changes in pulmonary MDA, SOD, and GSH levels when compared to control rats. 

### 2.4. DHM Downregulated NF-κB while Upregulating Nrf2/HO-1 Expression in Lung Tissues of MTX-Treated Rats

**Figure 3:**
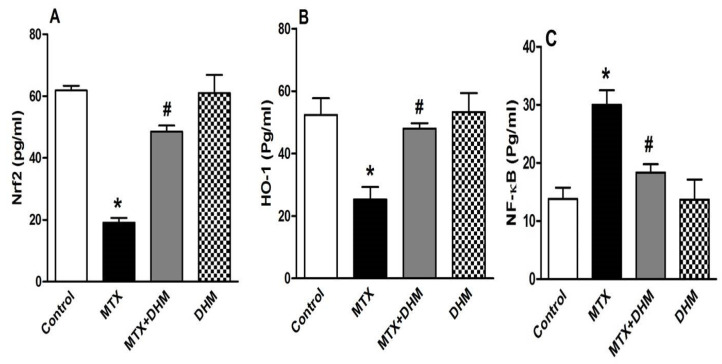
Effect of 2 weeks of treatment with dihydromyricetin (DHM, 300 mg/kg, p.o) on pulmonary levels of (**A**) Nrf2, (**B**) HO-1, and (**C**) NF-κB in methotrexate (MTX, 40 mg/kg, i.p)-induced lung toxicity. Data are presented as means ± SEM (n = 6). * and # indicate significant differences from the control and MTX groups, respectively, at (*p* < 0.05).

Nrf2 is a ROS-sensitive transcriptional factor that regulates the gene expression of antioxidant enzymes and enhances cellular resistance to ROS. The role of Nrf2 modulation in MTX-induced lung injury was assessed by measuring the pulmonary levels of Nrf2 and its downstream modulator, HO-1. As shown in Figure 3, compared to the control group, MTX administration was associated with a significant (*p* < 0.05) decline in the expression of Nrf2 (Figure 3A) and its downstream mediator, HO-1 (Figure 3B). Furthermore, MTX induced a profound inflammation of lung tissues, which was confirmed by the significant (*p* < 0.05) rise in NF-κB (Figure 3C). The latter is a nuclear factor that is essential for the production and release of inflammatory cytokines. This indicates that a crosstalk exists between Nrf2 and NF-κB in mediating MTX-induced lung injury. In contrast, administration of DHM with MTX significantly (*p* < 0.05) upregulated pulmonary Nrf2 and HO-1 levels (Figure 3A,B) while significantly (*p* < 0.05) attenuating NF-κB expression (Figure 3C) in lung tissues. Rats given DHM alone throughout the experiment showed non-significant changes in Nrf2, HO-1, and NF-κB expression when compared to control or vehicle-treated rats. 

### 2.5. DHM Alleviated the MTX-Induced Elevation of IL-1β and TGFβ-1 

**Figure 4:**
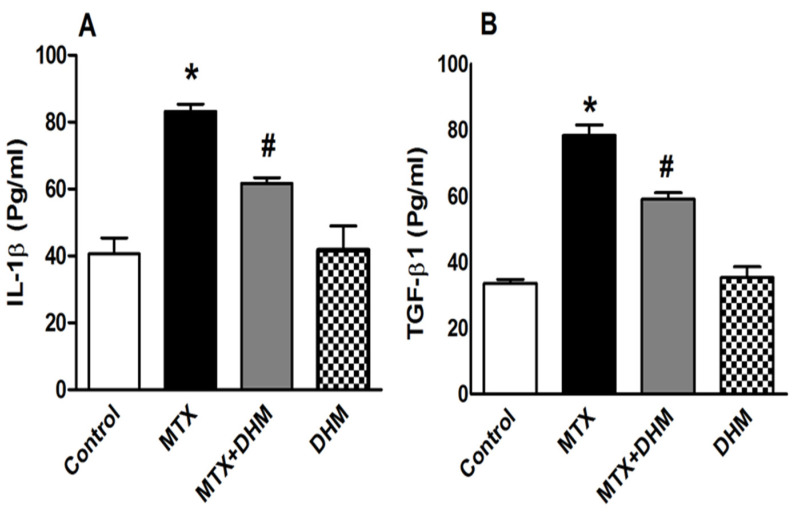
Effect of 2 weeks of treatment with dihydromyricetin (DHM, 300 mg/kg, p.o) on pulmonary levels of (**A**) IL-1β and (**B**) TGF-β1 in methotrexate (MTX, 40 mg/kg, i.p)-induced lung toxicity. Data are presented as means ± SEM (n = 6). * and # indicate significant differences from the control and MTX groups, respectively, at (*p* < 0.05).

As a consequence of the elevation of NF-κB in the MTX group, lung levels of the inflammatory mediator, IL-1β, were significantly (*p* < 0.05) increased (Figure 4A). Furthermore, MTX treatment resulted in a significant (*p* < 0.05) upregulation of TFG-β1 expression, which plays a central role in the development of tissue fibrosis (Figure 4B). Compared to MTX group, treatment with DHM significantly (*p* < 0.05) diminished the MTX-induced elevation of IL-1β and TGF-β1 in lung tissues (Figure 4A,B). The levels of IL-1β and TGF-β1 in the DHM group were not significantly different from the control group.

## 3. Discussion

The present study provides the first evidence for the protective effects of the natural flavonol DHM against MTX-induced lung toxicity. DHM abrogated MTX-induced interstitial pneumonitis and ameliorated the release of pro-fibrotic mediators in lung tissues via its antioxidant and anti-inflammatory properties. In the present study, we first reported a decline in body weight upon administration of a single dose of MTX (40 mg/kg). Several studies have attributed the MTX-induced weight loss to the development of gastrointestinal myositis, which may cause anorexia, impaired absorption, and diarrhea. The epithelial cells lining the gastrointestinal tract are rapidly replicating and highly susceptible to damage by cytotoxic drugs such as MTX [39,40]. Further, MTX can induce damage to many organs, including the lungs. MTX is thought to cause hypersensitivity pneumonitis, which is associated with the proliferation of lymphocytes and the recruitment of inflammatory cells, leading to alveolar damage [41]. In this study, the pulmonary histopathological examination and scoring demonstrated thickening of the alveolar walls, interstitial hemorrhage, alveolar consolidation, and massive infiltration with lymphocytes and eosinophils. Inflammatory cells contributed to the destruction of the alveolar epithelial lining and the development of pneumonitis. Our finding is in good agreement with an earlier study, which attributed the recruitment of inflammatory cells in lung tissues to MTX-induced release of eosinophil chemotactic factors from pulmonary fibroblasts and epithelial cells [16]. Additionally, MTX damages the vascular wall, leading to the extravasation of red blood cells. These subsequently attract mononuclear cells, leading to the activation of immune cells [15,42]. Peripheral eosinophilia was reported in nearly 50% of MTX-induced pneumonitis cases [16]. Notably, the type of inflammatory cells included was found to be dependent on either the dose or the length of exposure to MTX; neutrophils were common with low doses or short exposure to MTX, while lymphocytes were observed with high MTX doses or long exposure duration [41]. On the other hand, treatment with DHM for 2 weeks prevented the loss of body weight induced by MTX. However, the mean body weight of the control DHM group was not affected. Our findings indicate that DHM may be able to ameliorate the decrease in body weight caused by the anticancer drug adriamycin. This suggests a possible protective effect against gastrointestinal cytotoxicity. Importantly, DHM attenuated the MTX-induced pulmonary histopathological changes; few inflammatory cells were seen, and the alveolar tissue damage was minimal. This indicates a possible protective effect of DHM against MTX-induced lung damage. Likewise, DHM decreased pulmonary inflammation in lipopolysaccharide- and sepsis-induced acute lung injury models [37,38].

A large body of literature has emphasized the central role of ROS in MTX-induced lung cytotoxicity [19,43,44], as well as in other organs, including the liver, kidney, and intestine [9,45,46]. MTX increases the formation of free radicals, which are key features of DNA, protein, and cell membrane damage. Accumulated eosinophils, monocytes, and neutrophils in the lung tissues of MTX-treated rats, are likely to be associated with increased oxidative stress because they are major sources of ROS formation. Previous studies have identified mitochondrial damage as a major contributor to MTX-induced ROS overproduction. MTX induced renal and hepatic damage via disruption of the mitochondrial membrane potential, decreasing mitochondrial ATP and GSH levels and leading to increased mitochondrial permeabilization of ROS and cytochrome c. This further induces apoptotic cell death [47,48]. Accordingly, we reported an elevation of MDA levels in lung tissues upon MTX administration. MDA is a product of oxidative degradation of membrane phospholipids, as well as an indicative parameter of increased ROS release. Moreover, the pulmonary levels of GSH and SOD were minimized, either due to consumption with the excess ROS or suppression of antioxidant gene expression [47]. SOD is an enzymatic antioxidant that neutralizes superoxide anion radicals by converting them into hydrogen peroxide or molecular oxygen. GSH is a non-enzymatic antioxidant that acts as a ROS scavenger. Our data are compatible with previous findings, and our study confirms that oxidative stress, at least in part, participates in MTX-induced lung injury [18,19,43]. 

On the other hand, treatment with DHM induced a decline in MDA and an increase in pulmonary GSH and SOD levels. DHM is well-known to have antioxidant activity [29,33], and, thus, could attenuate MTX-induced hepatotoxicity via its antioxidant effects [9]. The antioxidant effects of DHM were shown to be partially dependent on upregulation of hemoxygenase-1 (HO-1) and activation of Nrf2 nuclear translocation [34].

Nrf2, a critical transcriptional factor for cell survival, enhances cellular resistance to oxidative damage and encodes for gene expression of antioxidant enzymes, including GSH, SOD, and catalase. Nrf2 is activated and translocated to the nucleus, then binds to antioxidant response elements (ARE) in order to induce the expression of antioxidant genes [49,50,51]. One of these genes is HO-1, which regulates antioxidant activities as well as inflammation, fibrosis, and apoptosis [52]. Herein, MTX administration attenuated Nrf2 and HO-1 expression, which led to increased oxidative stress in lung tissues. A similar link was found between Nrf2 downregulation and MTX-induced toxicity in other organs as the liver, kidneys, and testes [53,54,55,56]. Evidence has shown that MTX inactivates Nrf2 by inhibition of its translocation to the nucleus [57]. Based on these findings, we hypothesized that inactivation of the Nrf2/HO-1 pathway by MTX contributes to the development of oxidative stress-induced lung injury. 

On the contrary, co-treatment with DHM upregulated the expression of Nrf2 and HO-1 in lung tissues, increased antioxidant activity, and mitigated the pulmonary toxicity of MTX. The role of Nrf2 in positively regulating the antioxidant and redox hemostasis of DHM has previously been verified. DHM attenuated ethanol-induced hepatic injury, mitigated septic-induced renal damage, and protected endothelial cells against inflammatory damage and hyperglycemia-induced damage by activating Nrf2 signaling [36,58,59,60]. Based on the literature, DHM is able to activate Nrf2 signaling by enhancing the expression of P62, which binds to Keap-1 and forms a complex with it, leading to enhanced Keap-1 degradation. Keap-1 binds to Nrf2 in the cytoplasm; therefore, Keap-1 degradation enhances the release of Nrf2 and the subsequent nuclear translocation and activation [36,61]. Thus, the antioxidant effects of DHM are possibly mediated via Nrf2 activation. It is important to mention that Nrf2 activation can increase cellular resistance to damage by maintaining mitochondrial homeostasis. Nrf2 enhances mitochondrial biogenesis, i.e., replacement of dysfunctional mitochondria with new functioning ones, through stimulation of mtDNA replication [62,63]. Polyphenolic compounds, such as flavonoids, exhibit protective effects in different cell types through biogenesis of new mitochondria and clearance of dysfunctional ones [62,63]. More recently, DHM protected cardiomyocytes against rh-endostatin toxicity by alleviating mitochondrial damage [64]. Altogether, the cytoprotective effects of DHM are likely mediated through preservation of mitochondrial function. Future studies may be needed to elucidate the role of DHM-induced mitochondrial biogenesis against lung injury caused by MTX.

Another signaling pathway that may possibly be involved in MTX-induced inflammatory injury is the nuclear factor κB (NF-κB) [9,65,66]. NF-κB is a master regulator of a variety of genes involved in inflammatory responses and leads to marked production of proinflammatory cytokines, including IL-1, IL-8, IL-6, and TNF-alpha. Activation of NF-κB is triggered by different stimuli, such as ROS, inflammatory cells, and the proinflammatory cytokine IL-1β [67,68,69]. Herein, as well as in other studies, MTX promoted the expression of the nuclear factors κB (NF-κB) and IL-1β. The latter participates in the inflammatory response through the induction of leucocyte migration and accumulation at the injury site [18,19,44,70]. Furthermore, in the MTX-treated group, we noticed that elevated NF-κB expression was correlated to suppression of Nrf2 levels, suggesting a crosstalk between the two mediators [71]. Many studies reported a similar interplay; Nrf2 was found to suppress inflammation by negatively regulating the expression of IL-1β, IL-8, and IL-6, whereas decreased Nrf2 was associated with upregulation of NF-κB, leading to aggressive inflammation [72]. Additionally, HO-1, the regulator of Nrf2, inactivates NF-κB by inhibiting its nuclear translocation [73,74]. In a study using Nrf2 knockout mice, the lungs were massively infiltrated with lymphocytes and macrophages [75,76]. Our data are consistent with these findings since MTX administration caused downregulated Nrf2 and upregulated NF-κB, leading to marked lung inflammation. 

On the other hand, treatment with DHM reversed MTX-induced lung inflammation through downregulation of NF-κB and IL-1 β expression and upregulation of Nrf2. In vitro and in vivo studies have shown that attenuation of NF-κB signaling is a crucial pathway mediating the beneficial anti-inflammatory effects of DHM [33,73,77]. Recently, DHM was shown to protect against MTX-induced hepatotoxicity via suppression of NF-κB [9]. It was confirmed via research that DHM was able to inhibit NF-κB activation by binding directly to the I κB kinase and subsequently inhibiting IKK phosphorylation, which is an essential step in NF-κB activation. Moreover, DHM was able to attenuate TNF-alpha-mediated NF-κB activation [78,79,80].

Indeed, there is a debate regarding whether MTX induces fibrotic lung injury. In the present study, MTX caused an increased expression of transforming growth factor-β1 (TGF-β1), a profiberotic cytokine that is usually released in response to proinflammatory mediators and ROS [70,81]. Based on our findings, as well as those of others, the increase in TGF-β1 levels can likely be attributed to MTX-induced upregulation of NF-κB and suppression of Nrf2 [82,83]. Importantly, increased TGF-β1 is associated with the development of fibrotic lung changes brought on through mediation of the MTX-induced epithelial–mesenchymal transition (EMT). EMT is a pathophysiological state that promotes the conversion of damaged epithelial cells into collagen-forming myofibroblasts, which, in turn, cause excessive deposition of collagen in extracellular spaces and initiate lung fibrosis [84,85,86]. It is important to mention that suppressed Nrf2 levels, as well as increased IL-1β, may be involved in MTX-induced EMT [57]. Some studies have mentioned the role of Nrf2 in antagonizing EMT and inhibiting pulmonary fibrosis [87,88,89]. These results taken together, Nrf2 inactivation, as well as activation of TGF-β1, may partially participate in MTX-induced lung fibrosis. However, future studies are needed to confirm this. Herein, we determined that downregulation of TGF-1β is another cytoprotective mechanism of DHM against MTX-induced lung injury. DHM attenuated TGF-β1 expression through inhibition of oxidative stress and Nrf2 activation. Our data agree with the findings other studies which have reported the beneficial effects of DHM against lung, renal, and hepatic fibrosis [30,90,91]. 

Our study has shed light on DHM as a promising therapeutic agent which could be used to minimize MTX-induced pulmonary toxicity. Our previous study also reported the ability of DHM to ameliorate MTX-induced hepatotoxicity. Interestingly, DHM has been found to effectively relieve rheumatoid arthritis symptoms and mitigate the release of inflammatory mediators as TNF-α and interleukins through activation of the Nrf2/HO-1 pathway [35]. Additionally, in a collection of studies, it has been reported that DHM shows outstanding antitumor effects against breast cancer, lung cancer, hepatocellular carcinoma, and malignant melanoma [92]. Altogether, this evidence suggests that combining DHM with MTX may decrease the toxic effects of MTX without interfering with its therapeutic effects. On the contrary, DHM may act as an adjuvant therapy to MTX, allowing for the use of lower MTX doses and, thus, minimizing its side effects. Future studies are needed to confirm this. DHM is likely to have potential as an alternative to other drugs used to manage MTX toxicity in clinical application. 

## 4. Materials and Methods

An electronic search was carried out for the relevant information published in this work. The databases of Google Scholar, PubMed, Science Direct, Hindawi, and Web of Science were searched for articles published during the period from 1978 to 2023.

### 4.1. Drugs and Chemicals

DHM was purchased from Bulk DHM., London, UK. MTX (Mylan) 50 mg/2 mL vials were obtained from Haupt Pharma GmbH., Wolfratshausen, Germany. 

### 4.2. Animals and Experimental Design

In the present study, we used adult male Wistar rats (220–250 g). Rats were purchased from the National Research Center (Giza, Egypt) and were housed with 2 per cage at 25 °C. Rats were exposed to 12 h light and 12 h dark daily. Animals were kept for 2 weeks before starting the experiment in order to acclimatize them to the conditions. This study protocol followed the guidelines of the research ethics committee at the Faculty of Pharmacy, Minia University, Egypt (approval number: ES25/2020). To start the experiment, rats were randomly distributed into 4 groups (n = 6, each). Control group: rats in this group were given carboxymethyl cellulose, the vehicle of DHM, orally for 14 days, and on the 9th day of the experiment, they received a single intraperitoneal injection of saline, the vehicle of MTX. MTX group: rats received a single dose of MTX (40 mg/kg, i.p) on the ninth day of the experiment and 0.5% carboxymethyl cellulose orally for 14 days. MTX + DHM group: rats received oral DHM (300 mg/kg per day) dissolved in 0.5% carboxymethyl cellulose for 14 days, and on the 9th day of the experiment, they received a single intraperitoneal injection of MTX (40 mg/kg). DHM group: rats received oral DHM (300 mg/kg per day) dissolved in 0.5% carboxymethyl cellulose for 14 days, and on the 9th day of the experiment, they received an intraperitoneal saline injection. MTX and DHM doses were chosen according to previous studies [9]. At the end of the experiment, 24 h after the final treatment, all animals were weighed and then sacrificed under anesthesia. Blood samples were collected and centrifuged at 3000 rpm for 10 min to collect the sera. Both lungs were removed, cleaned of blood, dried, and weighed. The upper lobe of each left lung was cut and fixed in 10% neutral buffered formalin in order to prepare paraffin blocks for use in histopathological studies. The right lung was placed into liquid nitrogen and then kept at −80 °C. The lung tissue homogenates were made by homogenizing 20% *w*/*v* of lung tissues in 10 mM cold PBS buffer (pH = 7.4) containing protease inhibitor cocktail (Roche Diagnostics, Indianapolis, IN, USA). The homogenates were centrifuged at 6000 rpm for 15 min at 4 °C. The resulting supernatant was used for biochemical analysis. 

### 4.3. Histological Examination

Lung tissues were washed and fixed in 10% formal saline, processed for paraffin block formation, cut into sections 5 μm thick, and deparaffinized for hematoxylin and eosin staining [93] to evaluate the lung inflammation and tissue damage. Stained sections from all the studied groups were examined by Olympus microscope (Tokyo, Japan) and captured using a digital camera (ToupView, Zhejiang, China) controlled by ToupView software (version ×36, 3.5.563; Hangzhou ToupTek Photonics Co., Zhejiang, China). Measurements were performed in 10 non-overlapping fields for each group (n = 6) at a magnification power of ×40, as described [94]. 

Morphometric study: Lung injury scores were determined semi-quantitatively according to the following histological features: alveolar consolidation, thickness of alveolar wall, vascular congestion, interstitial hemorrhage, and inflammatory cell infiltration. Each field was recorded on a scale from 0 to 3 (0 = normal, 1 = mild damage, 2 = moderate damage, and 3 = severe damage); then, the mean score of each group for each observed histopathological alteration was determined as described previously [94,95]. 

### 4.4. Measurement of Oxidative Stress Parameters

The pulmonary oxidative stress was assessed by measuring the levels of malondialdehyde (MDA) in the lung tissue homogenates. MDA, the product of lipid peroxidation, is an indirect indicator of increased ROS production. The principle of this method is based on the reaction of MDA with thiobarbituric acid and the formation of adducts, which are measured colorimetrically [96]. Furthermore, in order to assess the antioxidant activity, commercially available kits obtained from (Biodiagnostics, Cairo, Egypt) were used for determination of the antioxidant glutathione (GSH, Catalog No. GR 2511) and the antioxidant superoxide dismutase (SOD, Catalogue No. SD 25 21) according to the manufacturer’s instructions.

### 4.5. Measurement of Nrf2, HO-1, NF-κB, IL-1β, and TGF-β1 Levels in Lung Tissues Using Enzyme-Linked Immunoassay (ELISA) Technique

First, the protein content in each sample was determined according to the method of Bradford, using the protein estimation kit (catalog# 2603300011730, Genei, Bangalore, India) according to the manufacturer’s instructions. The amount of protein used per lung tissue homogenate sample was 4 µg/µL. Then, rat ELISA kits were used to measure pulmonary levels of IL-1β (catalog number# SEA563Ra, Cloud-Clone Crop., Katy, TX, USA), Nrf2 (catalogue number# MBS3803826, Mybiosource, San Diego, CA, USA), HO-1 (catalogue number# ab279414, Abcam, MA, USA), NF-κB (catalogue number # CSB E13148r, Cusabio, Houston, TX, USA), and TGF-β1 (catalogue number # CSB-E04727r, Cusabio, Houston, TX, USA). All the measurement steps performed done based on the instructions of the manufacturer. 

## 5. Conclusions

The present study represents new evidence that dihydromyricetin exhibits protective effects against MTX-induced pulmonary toxicity. DHM restored the balance of Nrf2/NF-κB association, resulting in decreased release of ROS and inflammatory mediators. This eventually attenuated the activation of the profibrotic mediator TGF-β1. The multiplicity of actions of DHM through its antioxidant, anti-inflammatory, and antifibrotic effects suggests that DHM is a promising therapeutic candidate for preventing lung damage during MTX treatment.

## Figures and Tables

**Table 1:** Effect of MTX (40 mg/kg) and DHM (300 mg/kg) on mean body weight.

	Control (g)	MTX (g)	MTX + DHM (g)	DHM (g)
At 0 days	242.5 ± 11.09	230.0 ± 10.8	240.0 ± 9.13	226.3 ± 11.43
At 14 days	290.0 ± 9.13	196.5 ± 5.96 *	246.5 ± 6.24 ^#^	275.8 ± 6.3

Table 1 shows the mean body weight of each group (n = 6, each) at the beginning of the experiment (0 days) and at the end of the experiment (14 days). MTX: methotrexate (40 mg/kg, i.p), DHM: dihydromyricetin (300 mg/kg, p.o). *, significant compared to control group at *p* < 0.05, ^#^, significant compared to MTX-treated group at *p* < 0.05.

**Table 2:** Histopathological scoring of lung tissues (H&E sections) of control, MTX, DHM + MTX, and DHM groups.

Parameter	Groups			
	Control	MTX	MTX + DHM	DHM
Consolidation	0	1.8 *	0 ^#^	0
Interalveolar septa thickening	0	3.0 *	1.4 ^#^	0
Vascular congestion	0	3.3 *	1.0 ^#^	0
Interstitial hemorrhage	0	2.0 *	0.5 ^#^	0
Inflammatory cell infiltration	0	3.0 *	1 ^#^	0

Data are represented as the mean score of each group for each observed histopathological alteration. MTX: methotrexate (40 mg/kg, i.p), DHM: dihydromyricetin (300 mg/kg, p.o). 0 = normal, 1 = mild damage, 2 = moderate damage, and 3 = severe damage. *, significant compared to control group at *p* < 0.05; ^#^, significant compared to MTX-treated group at *p* < 0.05.

## Data Availability

Data is contained within the article.
